# FINANCIAL DIFFICULTY, HEALTH BEHAVIORS, AND MENTAL HEALTH OF FAMILY CAREGIVERS DURING THE COVID-19 PANDEMIC

**DOI:** 10.1093/geroni/igad104.2220

**Published:** 2023-12-21

**Authors:** Yujun Liu, M Courtney Hughes, Heng Wang

**Affiliations:** Northern Illinois University, DeKalb, Illinois, United States; Northern Illinois University, DeKalb, Illinois, United States; Rush University Medical Center, Chicago, Illinois, United States

## Abstract

The COVID-19 pandemic greatly burdened family caregivers in the United States and across the globe as they and their family members often suffered from the COVID-19 virus and its societal implications, like social isolation and a shift to remote healthcare. This study examines the impact of the COVID-19 pandemic on financial difficulty, health behavior, and mental health outcomes among family caregivers for older adults across the intersectionality of gender and race. Using the 2020 National Health and Aging Trends Study (NHATS) COVID-19 supplement for Family Members and Friends, our sample included adult family or friend caregivers of Medicare beneficiaries aged 65 or older in the US (N = 2,062). We modeled the interrelationships between financial difficulty, health behaviors, and mental health outcomes using a structural equation model. The results indicate that within the intersectionality framework, during the pandemic, female caregivers reported less vigorous activity, more changes in walking and sleeping patterns, and more eating and watching TV than before the pandemic compared to male counterparts. Compared to white caregivers, during the pandemic, non-white caregivers showed less vigorous activity and more changes in walking, sleeping, I eating, and TV watching patterns than before the pandemic. Financial difficulty was significantly and positively associated with negative mental health. Active behavior was significantly and negatively associated with negative mental health. Health professionals should consider the interrelationships between sociodemographic factors, physical activity levels, and financial difficulties that have come to light during the pandemic when designing future programs and policies to improve family caregiver health.

